# Supplementary material for: SARS-CoV-2 survival on skin and its transfer from contaminated surfaces
Source: PLoS One. 2025 Jun 20;20(6):e0325235. doi: 10.1371/journal.pone.0325235 (PMC12180721; doi:10.1371/journal.pone.0325235)
Supplement: S1 Fig — (DOCX) [file pone.0325235.s001.docx]

# SUPPORTING INFORMATION

# SARS-CoV-2 survival on skin and its transfer from contaminated surfaces

Ana K. Pitol ^1*^, Samiksha Venkatesan ^1^, Siobhan Richards ^1^, Michael Hoptroff ^2^, Amitabha Majumdar^2^, Grant Hughes ^1^

^1^ Liverpool School of Tropical Medicine, Departments of Vector Biology and Tropical Disease Biology, Centre for Neglected Tropical Diseases, Liverpool, L3 5QA, UK

^2^ Unilever Research and Development, Port Sunlight, CH63 3JW, UK

* Corresponding author

E-mail: ana.pitolgarcia@lstmed.ac.uk

### SARS-CoV-2 transfer from surface to skin

We compared the transfer efficiency of SARS-CoV-2 from surface (metallic rod) to human skin (ex-vivo skin, CTISkin) and to various skin models. The models include two full thickness 3D human skin models: LabSkin (LABSKIN) and EpiDermFT (MatTek) and one synthetic skin surrogate, Vitro-Skin (VITRO-SKIN). All the skins were maintained according to the manufacturer's instructions.

To perform the transfer events, we used the protocol described in the main article. Briefly, we added a droplet of 2μL of SARS-CoV-2 solution, containing ~10^7^ PFU/mL, to the centre of a metallic rod and allowed the inoculum to dry. After drying time (1 hour), we placed the skin (VitroSkin, LabSkin, MatTek, or ex-vivo skin) on a balance placed inside the biological safety cabinet and followed with a 10 second contact event (150 ± 20 gr) between the metallic rod and the skin. After the transfer, we recovered the virus on from the surface and the skin by pipetting up and down 15 times using culture media (DMEM supplemented with 2% FBS). After finishing with all the skin transfers, we serial dilute the samples and plated them. Samples were quantified using standard plaque assays. Transfer efficiency was estimated using the following formula:

$$TE (\%)=\frac{Virus Skin (PFU)}{Virus Surface (PFU) + Virus Skin (PFU)}$$

where $Virus Skin (PFU)$ and $Virus Surface (PFU)$ are the number of viruses recovered from the skin and from the surface after the transfer event.

**Results**: SARS-CoV-2 transfer to ex-vivo human skin was similar to transfer to LabSkin but different to the transfer to MatTek and Vitro-Skin (Figure S1). Based on these data, we selected Labskin as a model for human skin in the proposed experiments given its similarity to human skin explants.

**S1 Fig. Transfer efficiency of SARS-CoV-2 from surface to skin as a function of skin type.** The box plots show the distribution of transfer efficiency (TE), with the top and bottom edges representing the 25th and 75th percentiles, the centerline indicating the median value, and the whiskers extending to the highest and lowest observed values. The total number of transfer events was four for ex vivo skin and LabSkin, nine for MatTek, and five for VitroSkin. The sample size was determined by the availability of the different skin types. Experiments were conducted side-by-side to minimize variation in TE due to environmental factors such as humidity and temperature.
