# Supplementary material for: SARS-CoV-2 survival on skin and its transfer from contaminated surfaces
Source: PLoS One. 2025 Jun 20;20(6):e0325235. doi: 10.1371/journal.pone.0325235 (PMC12180721; doi:10.1371/journal.pone.0325235)
Supplement: S1 Table — (DOCX) [file pone.0325235.s002.docx]

# SUPPORTING INFORMATION

# SARS-CoV-2 survival on skin and its transfer from contaminated surfaces

Ana K. Pitol ^1*^, Samiksha Venkatesan ^1^, Siobhan Richards ^1^, Michael Hoptroff ^2^, Amitabha Majumdar ^2^, Grant Hughes ^1^

^1^ Liverpool School of Tropical Medicine, Departments of Vector Biology and Tropical Disease Biology, Centre for Neglected Tropical Diseases, Liverpool, L3 5QA, UK

^2^ Unilever Research and Development, Port Sunlight, CH63 3JW, UK

* Corresponding author

E-mail: ana.pitolgarcia@lstmed.ac.uk

**S1 Table. Virus transfer efficiency (TE, %) from surface to skin.**

This table presents the TE data that directly compares with the conditions used in this study, specifically dry transfer events, where the TE was estimated using a similar methodology than the one reported in the main manuscript.

| **Ref.** | **Virus** | **Surface** | **Skin** | **Drying**  **Time (min)** | **Contact time and contact pressure** | **Number of replicates** | **TE ± SD (%)** | **T and H** |
| --- | --- | --- | --- | --- | --- | --- | --- | --- |
| This study | Bacteriophage Phi 6 | Plastic | Volunteers’ fingers | 50 | 10 sec,  1,470 Pa | 50 | 11.5 ± 12.1 | 19-20 °C  26-30 % |
|  |  |  | Labskin | 52 |  | 52 | 12.2 ± 13.4 |  |
|  | SARS-CoV-2 | Plastic | Labskin | 24 |  | 24 | 13.7 ± 17.2 |  |
|  |  | Steel |  | 24 |  | 24 | 13.2 ± 27.6 |  |
|  |  | Cardboard* |  | 24 |  | 24 | 2.9 ± 9.6 |  |
| (1) | Bacteriophage MS2 | Steel | Volunteers’ fingers | 6 | 10 sec,  9,800 Pa | 6 | 37.4 ± 16 | 19-25 °C  40-65 % |
|  |  | Plastic |  | 6 |  | 6 | 79.5 ± 21.2 |  |
|  |  | Paper currency |  | 6 |  | 6 | 0.7 ± 0.5 |  |
| (2) | Bacteriophage MS2 | Glass | Volunteers’ fingers | 75 | 10 sec,  25,000 Pa | 75 | 25 ± 23 | 10-22 °C  40-65 % |
| (3) | Bacteriophage MS2 | Steel | Volunteers’ fingers | 30 | 10 sec,  2,450 Pa | 30 | 34 ± 12 | 21-22 °C  13-74 % |
|  |  | Plastic |  | 30 |  | 30 | 37 ± 14 |  |
|  | Bacteriophage Phi6 | Steel |  | 30 |  | 30 | 23 ± 19 |  |
|  |  | Plastic |  | 30 |  | 30 | 28 ± 23 |  |
| (4) | Bacteriophage PRD-1 | Steel | Volunteers’ fingers | NA | 10 sec,  NA | NA | 33.5 ± NA | NA |
|  |  | Plastic |  | NA |  | NA | 65.8 ± NA |  |
| (5) | Hepatitis A | Steel | Volunteers’ fingers | 6 | 10 sec,  9,800 and 1960 Pa | 6 | 22 ± 7 | 22 ± 2 °C  45 ± 5 % |
| (6) | Feline Calicivirus | Steel | Volunteers’ fingers | NA | 10 sec,  1960-3920 Pa | NA | 7 ± 1.9 | 24 ± 2 °C  45 % |

*Cardboard TE (%) reported here was evaluated at different drying time than plastic and steel.

T = Temperature, H = Humidity.

References:

1. Lopez GU, Gerba CP, Tamimi AH, Kitajima M, Maxwell SL, Rose JB. Transfer efficiency of bacteria and viruses from porous and nonporous fomites to fingers under different relative humidity conditions. Appl Environ Microbiol. 2013;79:5728–34.

2. Julian TR, Leckie JO, Boehm AB. Virus transfer between fingerpads and fomites. J Appl Microbiol. 2010;109:1868–74.

3. Anderson CE, Boehm AB. Transfer Rate of Enveloped and Nonenveloped Viruses between Fingerpads and Surfaces. Appl Environ Microbiol [Internet]. 2021 Oct 1 [cited 2022 Nov 20];87(22). Available from: https://journals.asm.org/doi/10.1128/AEM.01215-21

4. Rusin P, Maxwell S, Gerba C. Comparative surface-to-hand and fingertip-to-mouth transfer efficiency of gram-positive bacteria, gram-negative bacteria, and phage. J Appl Microbiol. 2002;93:585–92.

5. Mbithi JN, Springthorpe VS, Boulet JR, Sattar S a. Survival of hepatitis A virus on human hands and its transfer on contact with animate and inanimate surfaces. J Clin Microbiol. 1992;30:757–63.

6. Bidawid S, Malik N, Adegbunrin O, Sattar SA, Farber JM. Norovirus cross-contamination during food handling and interruption of virus transfer by hand antisepsis: experiments with feline calicivirus as a surrogate. J Food Prot. 2004;67(1):103–9.
